# Pro-inflammatory cytokines attenuate glucose-stimulated insulin secretion from INS-1E insulinoma cells by restricting mitochondrial pyruvate oxidation capacity – Novel mechanistic insight from real-time analysis of oxidative phosphorylation

**DOI:** 10.1371/journal.pone.0199505

**Published:** 2018-06-28

**Authors:** Jonathan Barlow, Thomas P. J. Solomon, Charles Affourtit

**Affiliations:** 1 School of Biomedical and Healthcare Sciences, University of Plymouth, Plymouth, United Kingdom; 2 School of Sport, Exercise and Rehabilitation Sciences, University of Birmingham, Birmingham, United Kingdom; 3 Institute for Metabolism and Systems Research (IMSR), University of Birmingham, Birmingham, United Kingdom; Tohoku University, JAPAN

## Abstract

Pro-inflammatory cytokines cause pancreatic beta cell failure during the development of type 2 diabetes. This beta cell failure associates with mitochondrial dysfunction, but the precise effects of cytokines on mitochondrial respiration remain unclear. To test the hypothesis that pro-inflammatory cytokines impair glucose-stimulated insulin secretion (GSIS) by inhibiting oxidative ATP synthesis, we probed insulin release and real-time mitochondrial respiration in rat INS-1E insulinoma cells that were exposed to a combination of 2 ng/mL interleukin-1-beta and 50 ng/mL interferon-gamma. We show that 24-h exposure to these cytokines dampens both glucose- and pyruvate-stimulated insulin secretion (*P* < 0.0001 and *P* < 0.05, respectively), but does not affect KCl-induced insulin release. Mirroring secretory defects, glucose- and pyruvate-stimulated mitochondrial respiration are lowered after cytokine exposure (*P* < 0.01). Further analysis confirms that cytokine-induced mitochondrial respiratory defects occur irrespective of whether fuel oxidation is coupled to, or uncoupled from, ATP synthesis. These observations demonstrate that pro-inflammatory cytokines attenuate GSIS by restricting mitochondrial pyruvate oxidation capacity. Interleukin-1-beta and interferon-gamma also increase mitochondrial superoxide levels (P < 0.05), which may reinforce the inhibition of pyruvate oxidation, and cause a modest (20%) but significant (P < 0.01) loss of INS-1E cells. Cytokine-induced INS-1E cell failure is insensitive to palmitoleate and linoleate, which is at odds with the cytoprotection offered by unsaturated fatty acids against harm caused by nutrient excess. Our data disclose a mitochondrial mechanism for cytokine-impaired GSIS in INS-1E cells, and suggest that inflammatory and nutrient-related beta cell failure emerge, at least partly, through distinct paths.

## Introduction

Impaired glucose-stimulated insulin secretion (GSIS) by pancreatic beta cells (β-cells) contributes to the hyperglycemic state that characterizes type 2 diabetes [1]. While certain conditions are strongly associated with this impairment, for example the excessive nutrient levels that circulate in obesity [2], the pathological mechanism of β-cell dysfunction in type 2 diabetes is incompletely understood. Several symptoms and complications of type 2 diabetes involve activation of the immune system and a consequent state of chronic low-grade inflammation [3,4]. Inflammation is associated with impaired β-cell function [4,5] and decreased β-cell mass [6,7]. The cause of β-cell inflammation in type 2 diabetes is debated, but likely relates to obesity as it is widely accepted that elevated nutrient levels stimulate the expression of interleukin-1β (IL-1β) in human pancreatic islets, via activation of the NLRP3 inflammasome [8–10] or the NF-κB pathway [11], which causes a pro-inflammatory state [12]. In turn, IL-1β upregulates numerous other cytokines and chemokines [13,14] and indeed reinforces its own expression causing a vicious cycle [15]. Chronic upregulation of pro-inflammatory cytokines is regarded a hallmark for impaired insulin secretion [5] and increased β-cell apoptosis [5] in the pathophysiology of type 2 diabetes.

The precise mechanisms by which cytokines alter β-cell function and mass have not been established conclusively. Cytokine-induced β-cell failure is likely mediated by nitric oxide (NO) that results from activation of inducible nitric oxide synthase [16], but NO-independent inflammatory mechanisms have also been suggested [17–19]. Cytokine-provoked NO may inhibit glycolysis [20–24] and/or the mitochondrial TCA cycle [25,26], but functional bioenergetic consequences of such inhibition have not been demonstrated to date. It is worth notice in this respect that NO may in fact benefit the bioenergetics of inflamed cells as it stimulates mitochondrial biogenesis under certain circumstances [27]. Moreover, it remains unclear if and how inflammatory GSIS defects relate mechanistically to β-cell failure caused by nutrient excess [28].

Mitochondria are essential for GSIS as glucose-fueled oxidative ATP synthesis causes a rise in the cytosolic ATP/ADP ratio, which triggers the electrophysiological events that are responsible for the eventual exocytosis of insulin-containing granules [29]. Moreover, β-cells fully depend on mitochondria to meet the high energy cost of insulin synthesis (*cf*. [30]) and exocytosis [31,32] because they are unable to make ATP via anaerobic glycolysis [29]. As mitochondrial dysfunction has indeed been associated with β-cell failure [33] and because mitochondrial targets of cytokine-induced NO have been reported [25,26], we set out to explore possible effects of pro-inflammatory cytokines on real-time mitochondrial activity of INS-1E insulinoma cells. Here we use IL-1β and interferon-gamma (IFN-γ) to model the inflammatory milieu in diabetes and show that these cytokines attenuate GSIS and dampen both glucose-sensitivity and coupling efficiency of oxidative phosphorylation. Concomitantly, these cytokines increase mitochondrial superoxide production and provoke a modest (20%) cell loss. Detailed real-time respiratory analysis reveals that IL-1β and IFN-γ impair ATP synthesis and, consequently, GSIS by restricting the mitochondrial capacity for oxidizing pyruvate.

## Materials and methods

### Cell culture

Rat INS-1E insulinoma cells (RRID: CVCL_0351), a well-established pancreatic beta-cell model [34] were maintained in RPMI-1640 medium containing 11 mM glucose, 5% (v/v) fetal bovine serum (FBS), 10 mM Hepes, 1 mM sodium pyruvate, 50 U/mL penicillin, 50 μg/mL streptomycin, 500 μM β-mercaptoethanol and 2 mM GlutaMAX [34]. Cells were seeded at 6 x 10^4^ cells/well and, at 70–80% confluence, exposed to a mixture of 2 ng/mL IL-1*β* and 50 ng/mL IFN-*γ*, in the absence or presence of non-esterified fatty acids (NEFAs), for 24 hr in RPMI medium containing 11 mM glucose. Notably, the applied cytokine concentrations are used throughout the literature [11,17,35–37], as they reflect pathological levels observed during the development of diabetes. Palmitoleate and linoleate were conjugated to bovine serum albumin (BSA) at molar ratios of 2:1 and 4:1, respectively, yielding free fatty acid levels of 20 nM as estimated assuming binding parameters reported by Huber *et al*. (2006) [38]. FBS was omitted from the growth medium for NEFA experiments.

### Insulin secretion

INS-1E cells seeded and exposed to cytokines ± NEFAs on 96-well culture plates were washed and starved for 1 h at 37 ^o^C under air in low-glucose Krebs-Ringer Hepes (KRH) medium comprising 135 mM NaCl, 3.6 mM KCl, 10 mM Hepes (pH 7.4), 0.5 mM MgCl_2_, 1.5 mM CaCl_2_, 0.5 mM NaH_2_PO_4_, 2 mM GlutaMAX, 2.5 mM glucose and 0.2% (w/v) BSA. Assay buffer was then replaced with fresh KRH medium and cells were incubated for 30 min on a shaking plate incubator (Labnet International, Oakham, UK) at 100 rpm. Next, supernatants were collected on ice and replaced with KRH containing either 20 mM glucose, 5 mM sodium pyruvate or 30 mM KCl. After another 30-min incubation, supernatants were collected on ice. Supernatants were centrifuged at 1,000*g* to pellet any detached cells and assayed for insulin by enzyme-linked immunosorbent assay (#10-1247-01, Mercodia, Sweden) or homogenous time-resolved fluorescence (#62IN1PEG, Cisbio Bioassays, France). Secreted insulin was normalized to cell density (see below).

### Mitochondrial respiration and superoxide

Mitochondrial respiration was measured in intact attached INS-1E cells as described in detail before [39]. Briefly, INS-1E cells seeded and treated with cytokines ± NEFAs on XF24 cell culture plates (Seahorse Bioscience, Agilent Technologies) were washed into KRH assay buffer and incubated for 50 min at 37 ^o^C under air. XF24 plates were then transferred to a Seahorse XF24 extracellular flux analyzer (controlled at 37°C) for a 10-min calibration and 4 measurement cycles to record basal cellular respiration. Glucose or sodium pyruvate were added at 20 and 5 mM, respectively, to stimulate respiration. Subsequently, 2 μg/mL oligomycin or 3 μM BAM 15 (Tocris Bioscience, Bristol, UK) and a mixture of 2 μM rotenone plus 2 μM antimycin A were added sequentially to, respectively, inhibit the ATP synthase or uncouple oxidative phosphorylation, and determine non-mitochondrial respiration. Superoxide was determined by monitoring MitoSOX and DHE (hydroethidine) oxidation in time as described before [40].

### Cell density

Density of attached cells was determined in 96-well plates by 4',6-diamidino-2'-phenylindole dihydrochloride (DAPI) fluorescence [40]. Cells seeded and treated with cytokines ± NEFAs were washed with phosphate-buffered saline (PBS) and fixed in 4% paraformaldehyde (ThermoFisher Scientific, #28908). After fixation, cells were washed again in PBS and then loaded with 0.5 *μ*g/mL DAPI. To limit background detection after loading with DAPI, cells were washed 4 times with PBS. DAPI fluorescence (λ_ex/em_ = 350/460 nm) was detected using a PHERAstar FS plate reader (BMG LABTECH) in fluorescence intensity, bottom-reading and well-scanning mode.

### Statistical analysis

Significance of mean differences was tested by unpaired Student’s t-tests or two-way ANOVA with Sidak multiple comparison analysis using GraphPad Prism Version 6.0 for Mac OS X (GraphPad software, San Diego, CA, USA). Data are presented as means ± SEM.

## Results and discussion

### Cytokines concomitantly impair GSIS and lower mitochondrial ATP synthesis

**Fig 1:**
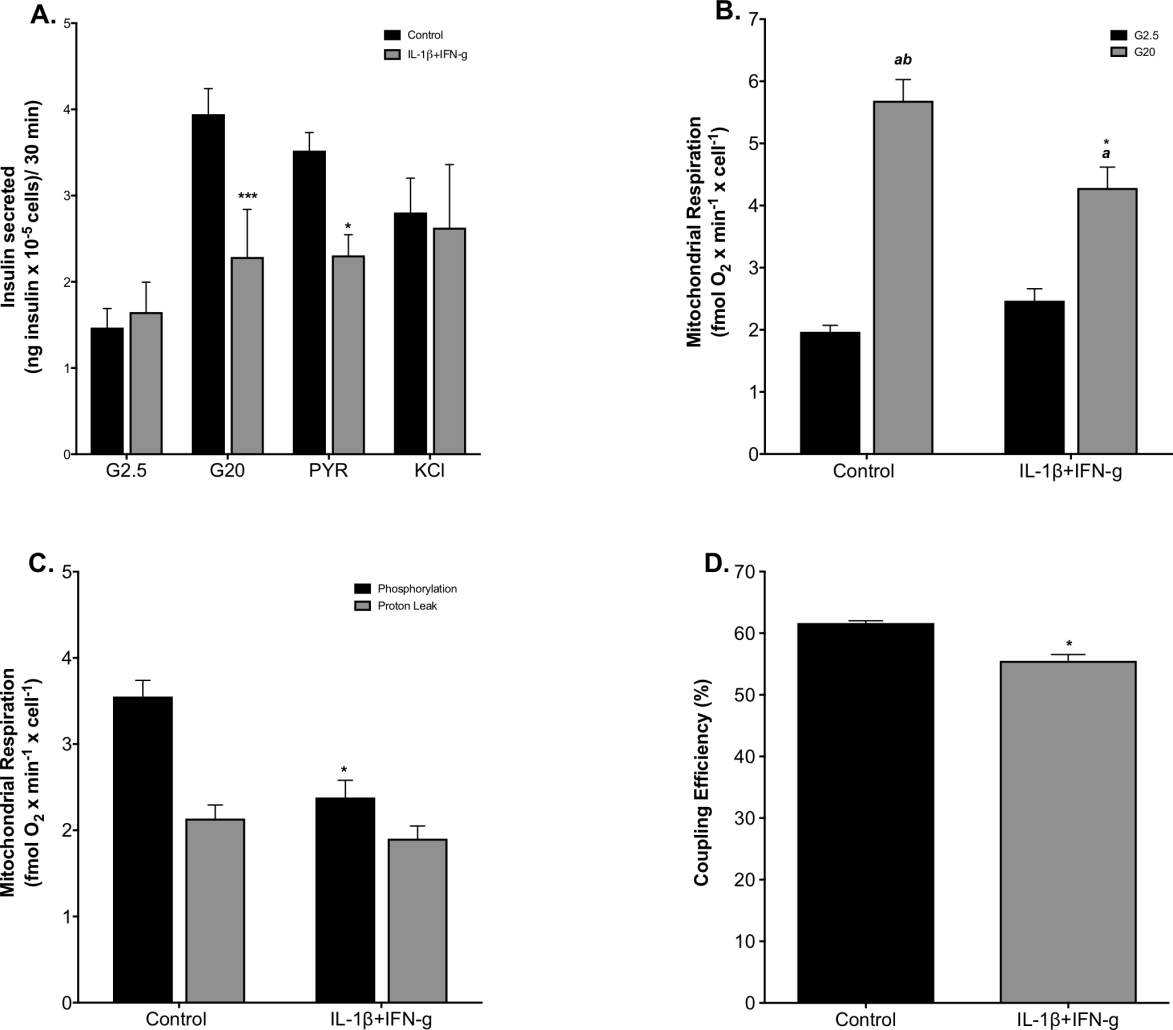
Cytokine-induced GSIS impairment and oxidative phosphorylation defects. Cells were grown in fully supplemented RPMI (black bars) or exposed for 24 h to a combination of 2 ng/mL IL-1β plus 50 ng/mL IFN-γ (grey bars). The rate of insulin secretion was normalized to cell number (A) and was measured at 2.5 mM glucose (G2.5), 20 mM glucose (G20), 5 mM sodium pyruvate (PYR) or 30 mM KCl. Absolute mitochondrial respiration normalized to cell number was measured ± 20 mM glucose (B)—grey and black bars, respectively. Glucose-stimulated respiration used to make ATP or associated with proton leak was determined as oligomycin-sensitive or oligomycin-resistant mitochondrial oxygen uptake, respectively (C)—black and grey bars, respectively. Coupling efficiency of oxidative phosphorylation was calculated as the percentage of glucose-stimulated oxygen uptake used to make ATP (D). Data are means ± SEM from 3–7 individual experiments (S1 Data) with each condition repeated 3–5 times. Statistical significance of mean differences was tested by 2-way ANOVA: asterisks indicate statistically significant differences from equivalent RPMI controls (*P < 0.05 and ***P < 0.001). ^a, ab^ differs from the low-glucose condition (P < 0.05 and P < 0.001, respectively).

INS-1E insulinoma cells retain many characteristics of primary β-cells, including glucose-sensing ability, which is why they are a widely used β-cell model [34]. In this study, INS-1E cells increased their insulin secretion rate 2.7-fold in response to 20 mM glucose, a stimulation that lowered to 1.4-fold when cells had been exposed to a mixture of IL-1β (2 ng/mL) and IFN-γ (50 ng/mL) for 24 h (Fig 1A). Cytokine attenuation of GSIS is owing to decreased insulin release at a stimulatory glucose level since basal insulin release was unaffected (Fig 1A), which is consistent with the deleterious effects of pro-inflammatory cytokines (including IL-1β, IFN-ɣ and TNF-α) on insulin secretion reported by others [21,41,42]. Notably, cytokine-induced GSIS impairment thus differs from nutrient-provoked secretory defects, which are predominantly owing to stimulated basal insulin release [43]. Pyruvate stimulates insulin secretion to the same extent as glucose, and does so in a cytokine-sensitive manner, whereas KCl-induced insulin secretion is unaffected by cytokines (Fig 1A). Pyruvate stimulation of beta cell activity is not exclusive to insulinoma cells, as insulin secretion by isolated mouse islets is increased to the same extent in response to methyl-pyruvate as to glucose [44]. These data thus demonstrate that secretory defects emerge downstream from glycolysis and upstream from β-cell electrophysiology. Therefore, we determined how cytokines affected glucose-fuelled oxidative phosphorylation. Mirroring the GSIS phenotype, mitochondrial respiration in cytokine-exposed cells was stimulated significantly less by glucose (1.7-fold) than respiration in control cells—2.8-fold stimulation–(Fig 1B). This significant loss of glucose sensitivity is owing to decreased respiration at a high glucose level (Fig 1B) and is consistent with GSIS data. Like basal insulin release, basal mitochondrial respiration was unaffected by cytokines (Fig 1B). To establish the proportion of glucose-stimulated respiration that is used to make ATP, we inhibited respiration with oligomycin. Fig 1C shows that the oligomycin-sensitive oxygen uptake rate was 3.6 fmol oxygen/min/cell on average and the oligomycin-insensitive rate was 2.4 fmol oxygen/min/cell. Notably, IL-1β and IFN-γ lowered oligomycin-sensitive respiration, i.e., oxygen consumption coupled to ATP synthesis, whereas they left oligomycin-resistant respiration, i.e, oxygen consumption linked to mitochondrial proton leak, unaffected (Fig 1C). Consequently, cytokine exposure lowers coupling efficiency of oxidative phosphorylation from 62 ± 0.4% to 56 ± 1.0% (Fig 1D). This coupling efficiency phenotype is statistically significant (P < 0.05) and shows that cytokine exposure decreases the proportion of mitochondrial respiration used to make ATP [45]. Cytokine-induced impairment of GSIS thus coincides with dysfunctional oxidative phosphorylation, which suggests that deficient mitochondrial ATP synthesis is responsible for the insulin secretory defect.

### Cytokines restrict mitochondrial pyruvate oxidation capacity

**Fig 2:**
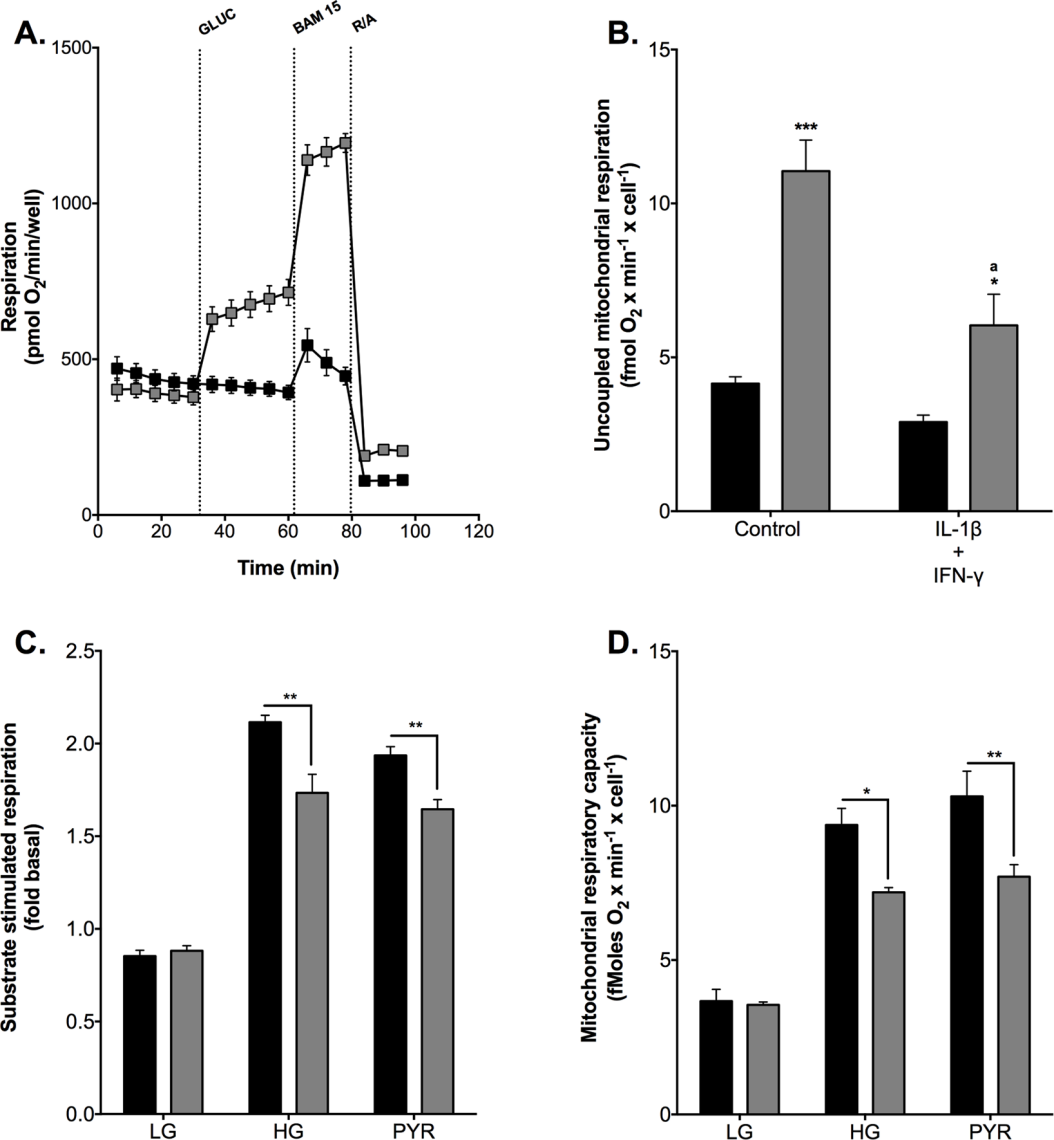
Cytokine-induced restriction of mitochondrial pyruvate oxidation. **(**A) mean respiratory traces illustrating the effect of glucose on coupled and uncoupled oxygen uptake. Cells were incubated in glucose-free KRH and then subjected to 20 mM glucose after 5 basal respiratory measurements (grey symbols)–the glucose level was not changed in control cells (black symbols). Subsequently, respiration was stimulated with 3 μM BAM 15 and then inhibited with a mix of 2 μM rotenone and 2 μM antimycin A (R/A). (B) cells were grown in fully supplemented RPMI (control) or exposed for 24 h to 2 ng/mL IL-1β plus 50 ng/mL IFN-γ. Mitochondrial respiration was uncoupled with 3 μM BAM 15 in the presence or absence of 20 mM glucose (grey and black symbols, respectively). Panels (C) and (D) cells were grown in full RPMI (black bars) or were exposed to 2 ng/mL IL-1β plus 50 ng/mL IFN-γ (grey bars). Respiration was stimulated with 20 mM glucose (HG) or 5 mM sodium pyruvate (PYR) in the absence (C) or presence (D) of 3 μM BAM 15, and substrate responses were normalized to basal oxygen uptake measured without added substrate–control cells were not stimulated by fuel (LG). Data are means ± SEM from 3 individual experiments (S1 Data) with each condition repeated 3–4 times. Mean differences were tested for statistical significance by 2-way ANOVA. (B) *^,^***differs from the no-glucose condition (*P* < 0.05 and *P* < 0.001, respectively); ^a^differs from high-glucose control condition (*P* < 0.01). Panels (C) and (D) *^,^**cytokine and control conditions differ (P < 0.05 and *P* < 0.01, respectively).

To better understand how cytokines inhibit mitochondrial ATP synthesis, we probed their effect on oxidative phosphorylation in more detail. Interestingly, glucose stimulation of respiration appeared most pronounced in the presence of BAM 15 (Fig 2A), a protonophore that uncouples oxidative phosphorylation. Uncoupled mitochondrial oxygen uptake is not controlled by ATP synthesis or ATP turnover, and thus generally reflects the *capacity* of cells for oxidizing fuel. The fuel oxidation capacity of INS-1E cells thus appears limited by the concentration of glucose (Fig 2B), which sits well with the glucose sensitivity of oxidative phosphorylation of pancreatic β-cells [46,47]. Cytokine exposure inhibits both coupled and *uncoupled* mitochondrial respiration (Fig 2B), which shows the respiratory inhibition emerges from a negative effect on glucose oxidation. Identical data were obtained when respiration was stimulated with pyruvate instead of glucose (Fig 2D). Pyruvate increases coupled (Fig 2C) and uncoupled (Fig 2D) mitochondrial oxygen consumption to the same extent as glucose, and the cytokine sensitivity of stimulated respiration is independent of fuel type Fig 2C and 2D. These respiratory data show that cytokines impair INS-1E cell function through inhibition of pyruvate oxidation, which is consistent with our GSIS data (Fig 1). The data rule out that IL-1β and IFN-γ exerted inflammatory effects upstream from mitochondria under the exposure conditions we applied.

### Cytokines increase mitochondrial superoxide and cause cell loss

**Fig 3:**
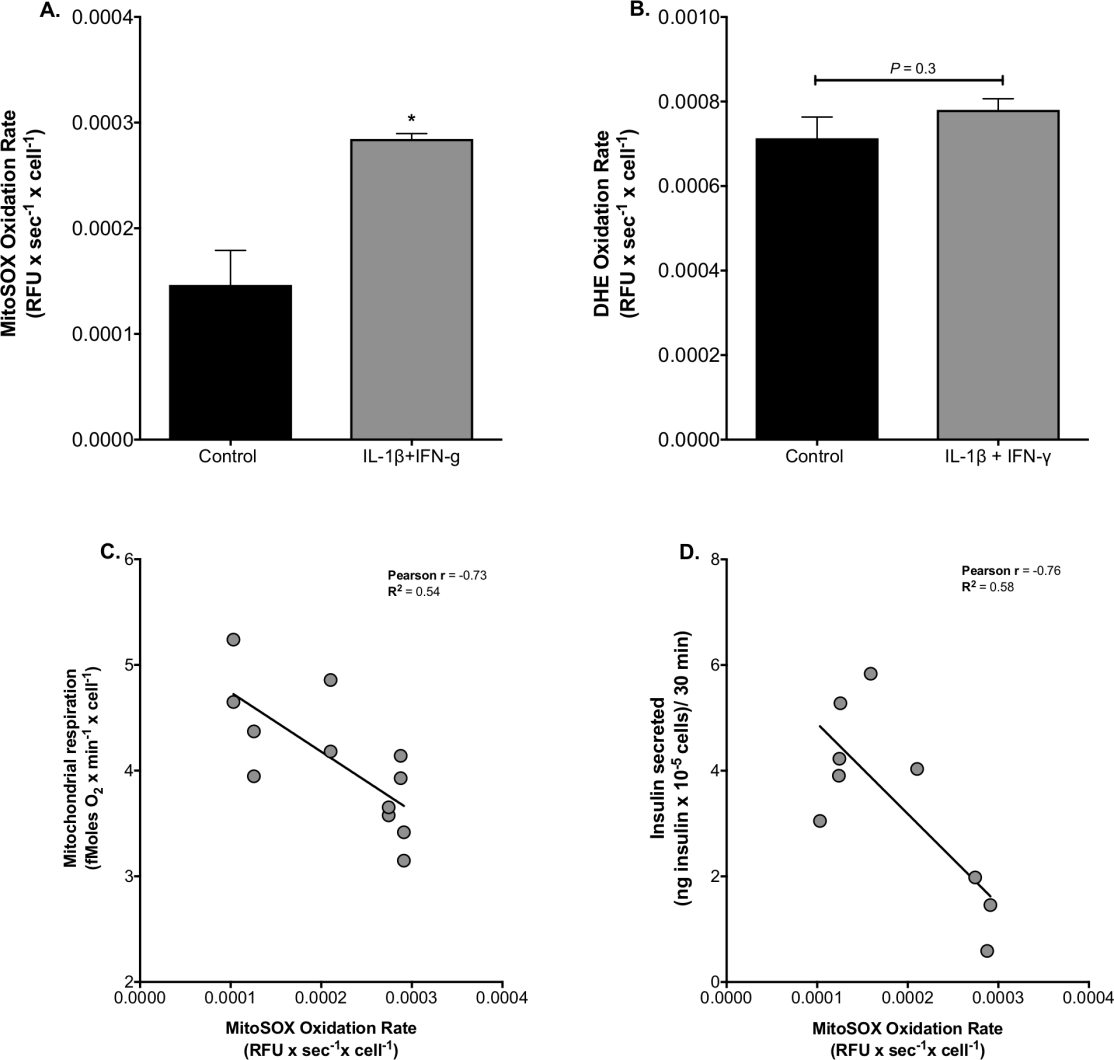
Cytokine-induced mitochondrial superoxide. (A) cells were grown in full RPMI (black bars) or were exposed for 24 h to 2 ng/mL IL-1β plus 50 ng/mL IFN-γ (grey bars). MitoSOX (A) and DHE (B) oxidation rates were measured without added glucose as described before [40] and the presented data are means of 3 independent experiments with each condition repeated 4–5 times. Mean differences were tested for statistical significance by unpaired Student's t-tests: *differs from the control condition (*P* < 0.05). Glucose-stimulated mitochondrial respiration (C) and glucose-stimulated insulin secretion (D) correlate inversely with the MitoSOX oxidation rate as confirmed by Pearson correlation analysis (P < 0.01 and P < 0.05, respectively). Data are stand alone repeats and were collected from 3 independent experiments (S1 Data).

**Fig 4:**
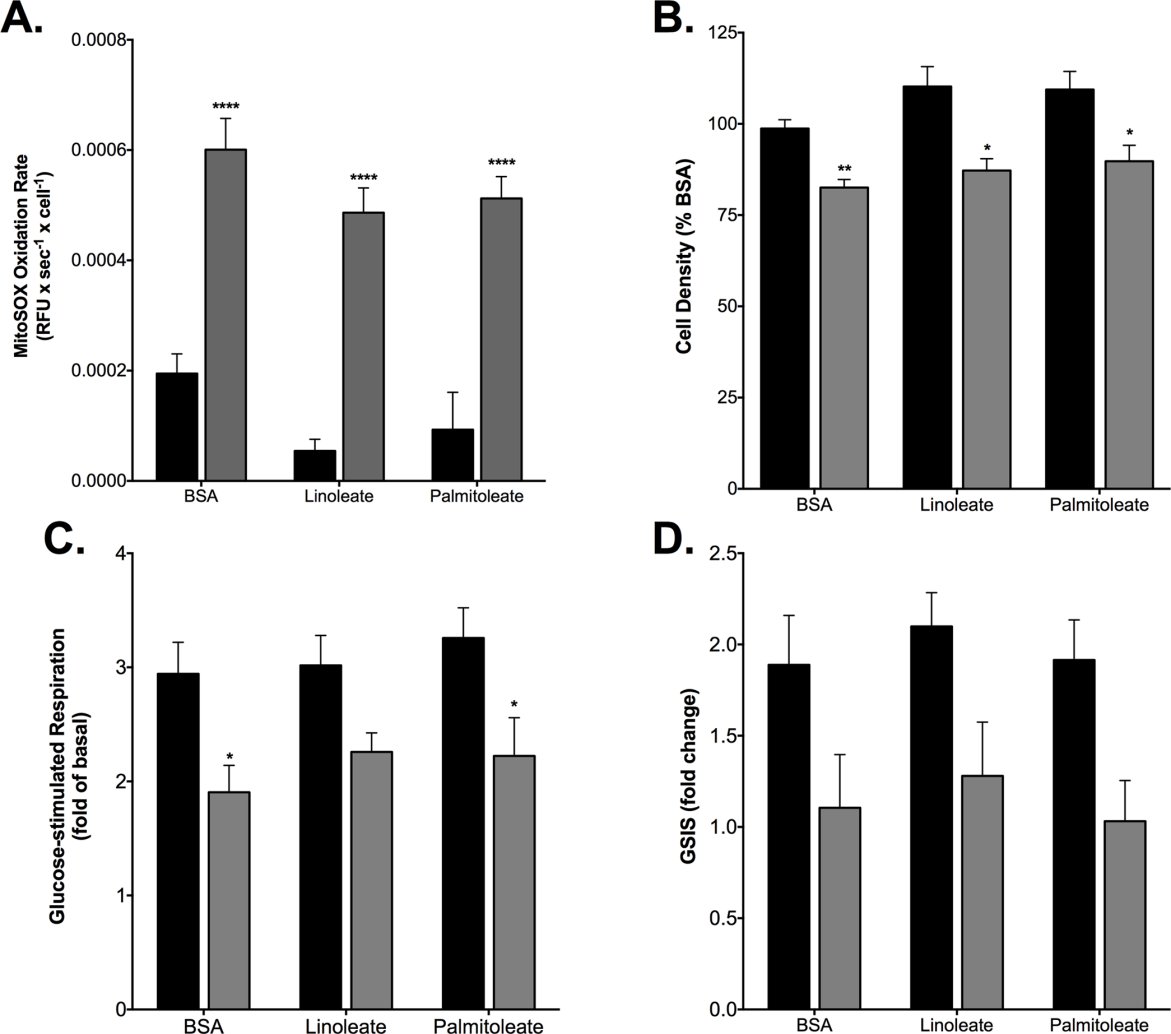
Unsaturated NEFAs do not protect against cytokine-induced β-cell failure. Cells were exposed for 24 h to 2 ng/mL IL-1β plus 50 ng/mL IFN-γ (grey bars) in serum-deprived RPMI containing BSA-conjugated linoleate or palmitoletae, or BSA alone. Control cells were incubated in the same media lacking the cytokines (black bars). NEFA effects were determined on MitoSOX oxidation rate (A)—normalized to cell number), cell density (B)—normalized to BSA control not exposed to cytokines, glucose-stimulated coupled mitochondrial respiration (C)—normalized to coupled respiration without added glucose and GSIS (D). Data are means ± SEM of 4 independent experiments (S1 Data) with each condition repeated 5 times. Mean differences were tested for statistical significance by 2-way ANOVA: *^,^**^,^****differs from the control condition (P < 0.05, P < 0.01 and P < 0.0001, respectively).

Pro-inflammatory cytokines are known to provoke production of reactive oxygen species (ROS) in β-cells [48] and, consistently, IL-1-β plus IFN-γ exposure increases the rate at which INS-1E cells oxidize MitoSOX ~ 2-fold (Fig 3A). MitoSOX is a mitochondria-targeted hydroethidine(DHE)-based probe that is widely used to detect superoxide, although it should be noted that the probe can also be oxidized by hydrogen peroxide (in the presence of peroxidases) and intracellular oxidases [49]. It is conceivable that cytokine-induced mitochondrial ROS are owing to the restricted pyruvate oxidation capacity. Oxidation of non-targeted DHE is not affected by cytokine exposure (Fig 3B). Glucose-stimulated mitochondrial respiration (Fig 3C) and glucose-stimulated insulin secretion (Fig 3D) both correlate inversely with MitoSOX oxidation rate, suggesting the possibility that cytokine-induced superoxide reinforces the inhibition of pyruvate oxidation that accounts for GSIS impairment. The cytokine effect on MitoSOX oxidation (Fig 3A) is reminiscent of that induced by NEFAs: 24-h palmitate exposure, against a high-glucose background, increases mitochondrial superoxide, an effect that correlates strongly with the loss of INS-1E cells and, notably, is largely prevented by palmitoleate, the monounsaturated counterpart of palmitate [40,50]. In contrast, unsaturated NEFAs do not attenuate cytokine-stimulated superoxide, as neither linoleate nor palmitoleate significantly lowers the cytokine-provoked MitoSOX oxidation rate seen in BSA-treated control cells (Fig 4A). Exposure to IL-1-β plus IFN-γ causes a 20% decrease of cell density (Fig 4B), which is likely owing to ROS-induced apoptosis [51,52], however we cannot exclude other mechanisms of cytokine-induced beta-cell death which have also been suggested [53,54]. This 20% decrease is small compared to that caused by palmitate [40,50] and, importantly, is not prevented by linoleate or palmitoleate (Fig 4B). Together, these data suggest that cytokine-induced generation of ROS and associated loss of cell density are mechanistically unrelated to palmitate-induced changes in ROS production. Cytokine inhibition of mitochondrial respiration (Fig 4C) and insulin secretion in response to 20 mM glucose (Fig 4D) are also not influenced by linoleate or palmitoleate.

## Discussion

Pro-inflammatory cytokines have well-established detrimental effects on the function and viability of pancreatic β-cells [4,35,52,55]. Our detailed real-time respiratory analysis reported here reveals functional consequences of cytokine exposure for β-cell oxidative phosphorylation, and highlights INS-1E cells as powerful model of β-cell bioenergetics. Previously, we predicted a dual role of mitochondrial uncoupling protein-2 in GSIS regulation and oxidative stress protection [56], which has proven correct in pancreatic islets [57,58]. Moreover, toxic nutrient effects on the bioenergetic behaviour of INS-1E cells align closely with those observed in islets [43]. Our current findings offer novel insight in the effects of cytokines on real-time mitochondrial function in β-cells and conclusively link a restricted pyruvate oxidation capacity to cytokine-impaired GSIS. Moreover, our results dissociate inflammatory defects from palmitate-induced defects, and provide clues as to how unsaturated NEFAs may protect against nutrient-induced β-cell damage.

### Mechanism of cytokine-induced GSIS impairment

**Fig 5:**
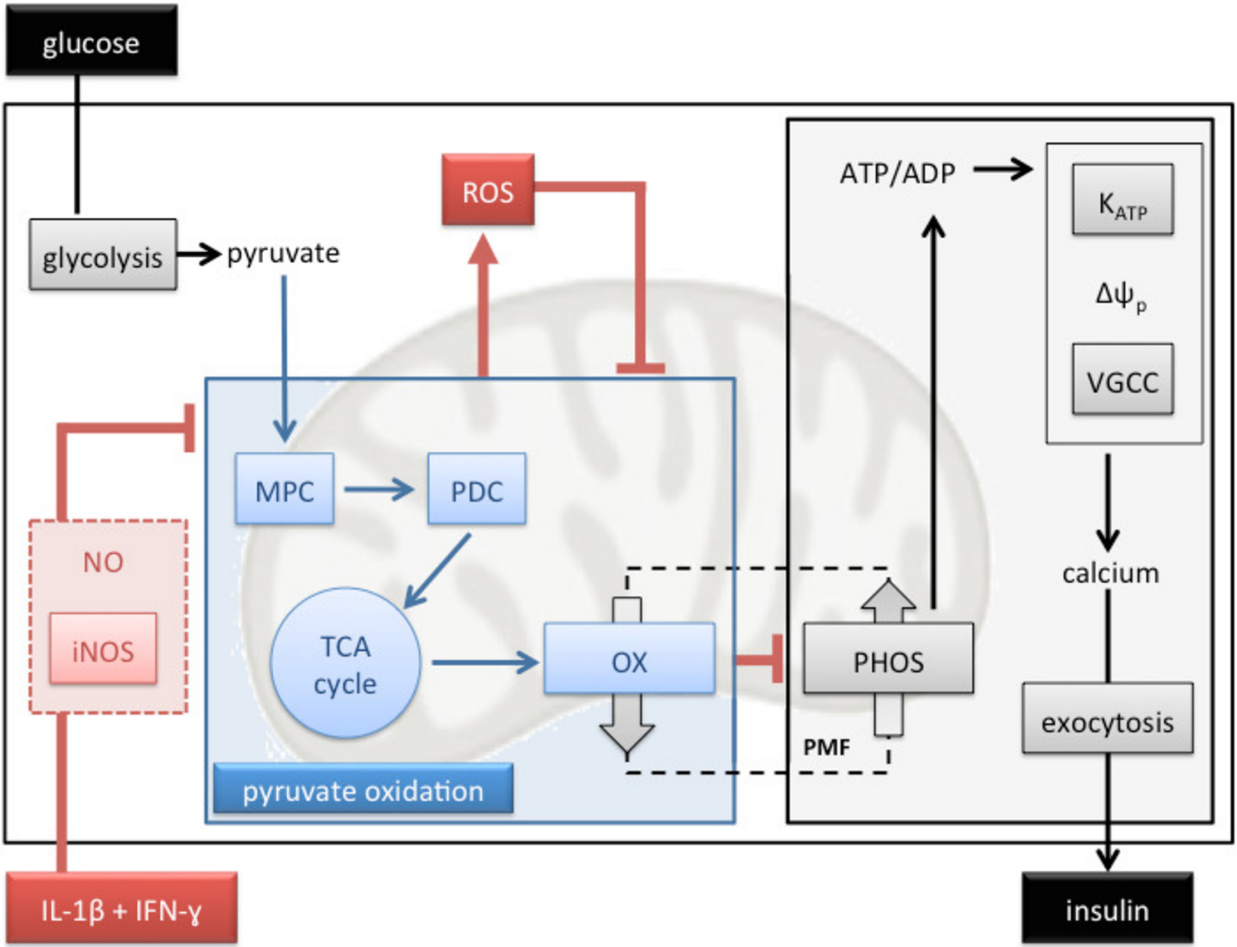
Mechanistic model of cytokine-induced GSIS impairment. Pancreatic β-cells respond to a rise in the blood glucose level by increasing their oxidative metabolism. Pyruvate that results from increased glycolysis is imported to mitochondria by the mitochondrial pyruvate carrier (MPC) and then oxidized by the pyruvate dehydrogenase complex (PDC) to acetyl-CoA, which fuels the tricarboxylic acid (TCA) cycle. Reducing power generated by these processes is oxidized by the mitochondrial electron transfer chain (OX) that conserves liberated energy in a protonmotive force (PMF), which drives ADP phosphorylation (PHOS). The consequent rise in the cytosolic ATP/ADP ratio inhibits ATP-sensitive potassium channels (K_ATP_), decreases the plasma membrane potential (Δψ_p_), opens voltage-gated calcium channels (VGCC), causes Ca^2+^ influx, and triggers exocytosis of insulin-containing granules. Exposure to IL-1β plus IFN-γ inhibits mitochondrial pyruvate oxidation (blue box) likely via activation of inducible nitric oxide synthase (iNOS) and consequent NO formation. Inhibition of pyruvate oxidation dampens bioenergetic glucose sensitivity and thus attenuates GSIS. Cytokine-induced reactive oxygen species (ROS) are a plausible consequence of inhibited pyruvate oxidation and may reinforce this inhibition.

Mitochondrial ATP synthesis is crucial for nutrient-secretion coupling in β-cells as bioenergetic fuel sensitivity is responsible for the glucose-induced rise in the cytosolic ATP/ADP ratio that triggers insulin secretion [47]. Our functional respiratory data reveal that IL-1β plus IFN-γ impair GSIS by restricting mitochondrial capacity for oxidising pyruvate (Fig 2), which is consistent with a well-established aconites inhibition by cytokine-induced NO [25,26]. Reported glucokinase inhibition [20–24] unlikely accounts for the inflammatory GSIS phenotype we report here (Fig 1A) as glucose- and pyruvate-stimulated electron transfer capacity are equally sensitive to IL-1β plus IFN-γ. Restricted pyruvate oxidation capacity dampens glucose sensitivity (Fig 2B) and coupling efficiency of oxidative phosphorylation (Fig 2C), and thus likely prevents an increase in the ATP/ADP ratio that initiates the electrophysiological events that are necessary for Ca^2+^ influx and eventual exocytosis of insulin-containing granules. Indeed, this pathological order of events (Fig 5) agrees with the lack of cytokine effect on KCl-induced insulin secretion (Fig 1A). The mechanism by which IL-1β plus IFN-γ inhibit pyruvate oxidation cannot be concluded directly from our data, but NO-involvement is likely [16,59]. Indeed, aconitase inhibition by NO [25,26] is expected to lower pyruvate oxidation as is NO inhibition of cytochrome *c* oxidase [60]. Moreover, cytokine-induced superoxide (Fig 3), which could arise from several sites [61] following increased reduction of the pyruvate oxidation apparatus, may react with NO to form peroxynitrite [62] that could exacerbate inflammatory respiratory dysfunction and consequent GSIS impairment (Fig 5). Our mitochondrial superoxide data (Fig 3) are consistent with a well-established ROS involvement in cytokine-induced beta cell failure [21,63–65]. Worth notice, it has been suggested that superoxide may in fact *ameliorate* cytokine-provoked β-cell dysfunction by converting NO to peroxynitrite [66].

### Palmitate and cytokine phenotypes are mechanistically distinct

Circulating NEFAs and cytokines are considered important molecular mediators that link obesity to type 2 diabetes [12] through mechanisms that are possibly related. Specifically, nutrient excess that characterizes obesity has been shown to stimulate the production of pro-inflammatory cytokines in human islets by NEFA-binding to β-cell toll-like receptors [67] and consequent upregulation of NF-κB signaling [12]. Nutrient surplus further causes β-cell inflammation by activating the NLRP3 inflammasome [12]. Although IL-1β plus IFN-γ indeed cause dysfunction and loss of INS-1E cells that appear similar to palmitate-induced defects [40,43,50,68], our data reveal that the underlying mechanisms are different. Both cytokines and palmitate cause oxidative phosphorylation defects, but attenuation of GSIS *coincides* with deficient ATP synthesis in the case of inflammatory stress (Fig 1), whereas it *precedes* mitochondrial dysfunction in the case of nutrient stress [43]. Furthermore, restricted oxidative capacity appears the sole cause of cytokine-impaired GSIS (Fig 5), whereas the deleterious palmitate GSIS phenotype is largely owing to stimulation of basal insulin secretion, most likely secondary to electrophysiological defects [43]. Cytokine effects on electrophysiology and insulin exocytosis are unlikely in our experiments as KCl-induced insulin secretion from INS-1E cells was not affected by IL-1ß and IFN-γ (Fig 1A). In agreement with [69], we also reveal that palmitate and cytokines stimulate mitochondrial superoxide production via distinct mechanisms, since palmitate-induced oxidative stress and consequent loss of INS-1E cells are prevented by palmitoleate [40,50], whilst equivalent cytokine phenotypes are unaffected by palmitoleate and linoleate (Fig 4).

### Protection against nutrient-induced β-cell failure

It is generally unclear how unsaturated NEFAs protect against damage caused by their saturated counterparts [70]. As discussed above, the cytokine defects we report here are *not* prevented by unsaturated NEFAs, which disagrees with observations published by others [35]. This disagreement may arise from experimental differences, which would suggest that protection by unsaturated NEFAs against inflammatory cell stress is not particularly robust. Because the design of our palmitate and cytokine experiments is comparable, the differential protection by unsaturated NEFAs against distinct oxidative stress mechanisms offers clues as to how palmitoleate prevents palmitate-induced cell loss. Specifically, our findings reveal that palmitoleate does not prevent ROS from triggering apoptosis, and, therefore, that protection must arise during ROS production. Both palmitate [40,50] and cytokines Figs 3A and 4A stimulate mitochondrial superoxide, but by distinct mechanisms given the differential palmitoleate effect on the rate of MitoSOX oxidation. Our cytokine exposures likely stimulate superoxide generation from one or more of the mitochondrial sites that are involved in (restricted) pyruvate oxidation (Fig 5), which include the pyruvate dehydrogenase complex and respiratory complexes I, II and III [61]. Palmitate exposure is expected to increase ROS production from the same sites against a background of high glucose [71], but, in addition, may cause superoxide production from the electron-transferring flavoprotein [61], which reduces the mitochondrial electron transfer chain during fatty acid β-oxidation. We speculate that unsaturated NEFAs may lower ROS production from this site.

## Conclusion

Our results identify pyruvate oxidation defects as an early step in cytokine-induced GSIS impairment in INS-1E cells. Given the previous consistency between bioenergetic effects in INS-1E cells and pancreatic islets (*cf*. papers cited above), the reported data suggest the mitochondrial electron transfer chain as a possible target for therapeutic prevention of pro-inflammatory loss of β-cell function and mass. As palmitate- and cytokine-induced defects arise via distinct mechanisms, multiple pharmacological interventions may be necessary to manage β-cell failure in obesity. Although the mechanism by which unsaturated NEFAs protect against obesity-related β-cell loss and dysfunction remains to be elucidated, our data suggest that their use against inflammatory stress is limited.

## Supporting information

S1 Data Excel data spreadsheet. Excel spreadsheet containing all relevant individual data values that were used to generate mean averages and standard errors for all data figures presented throughout this manuscript.
(XLSX)
